# Identification of actin mutants with neurodegenerative disease-like phenotypes via mutagenesis of the actin-ATP interface

**DOI:** 10.3389/fncel.2025.1543199

**Published:** 2025-06-04

**Authors:** Noah Mann, Keerthana Surabhi, Josephine Sharp, Mary Phipps, Maelee Becton, Jahiem Hill, Davis Roberts, Erzsebet M. Szatmari, Robert M. Hughes

**Affiliations:** ^1^Department of Chemistry, East Carolina University, Greenville, NC, United States; ^2^Department of Physical Therapy, East Carolina University, Greenville, NC, United States

**Keywords:** neurodegeneration, cytoskeleton, cofilin-actin rods, Hirano bodies, Cry2/CIB1, cellular stress

## Abstract

Cofilin-actin rods are a well-documented stress response in neuronal cells and their persistence is frequently associated with neurodegenerative disease. However, the role of specific actin residues in promoting the formation of cofilin-actin rods and other anomalous cytoskeletal structures is largely unknown. As it is increasingly suspected that specific mutations and post-translation modifications of actin may promote neurodegenerative disease, characterizing the role of these residues in cytoskeletal dysregulation is highly relevant. In this study, we focus on the actin-ATP interface, which has been proposed as a key mediator of cofilin-actin rod formation and the propensity of actin to respond to cellular stress. Using a light and stress-gated reporter of cofilin-actin cluster formation, we determine the impact of mutants associated with Actin-ATP binding on the propensity of actin to form anomalous structures in the presence and absence of applied cellular stress. This study identifies actin mutants that promote anomalous actin inclusions in HeLa cells and characterizes the manifestation of these phenotypes in cortical neurons. Mutations to the ATP phosphate tail-binding region of actin (K18A, D154A, G158L, K213A) were found to be particularly disruptive to actin phenotypes, and in several instances promote disease-associated actin-rich structures such as cofilin-actin rods and Hirano bodies. We find that these mutant phenotypes are largely consistent between cell types and display highly unusual inclusions in cultured cortical neurons, without leading to nuclear fragmentation and apoptotic death of the transfected cells. These mutants strengthen the association of residue-specific changes in actin with large-scale phenotypic and functional changes in the cytoskeleton, further implicating them in neurodegenerative disease progression.

## Introduction

Cytoskeletal dysregulation is a common feature of neurodegenerative diseases, including Alzheimer’s disease, Parkinson’s disease, and Huntingtin’s disease (Bamburg et al., 2010). Aberrant actin-rich structures are the byproducts of dysregulation and have consequences for cellular function and viability (Schmid et al., 2024). Cofilin-actin rods form under neurodegenerative disease-associated oxidative and energetic stress conditions such as ATP depletion, glutamate excitotoxicity, or a highly oxidative environment (Bamburg et al., 2010; Wurz et al., 2022). Accumulation and persistence of cofilin-actin rods in neurons can lead to the loss of dendritic spines and, therefore, the elimination of excitatory synapses. As a result, a deeper understanding of the structural factors contributing to the stress-associated interaction of cofilin and actin is critical for defining the biological underpinnings of neurodegenerative diseases (Bamburg and Bernstein, 2016; Bamburg and Bloom, 2009; Davis et al., 2009; Davis et al., 2011; Maloney and Bamburg, 2007; Minamide et al., 2000).

The actin-ATP interaction is at the core of stress-associated anomalous cytoskeletal structures such as cofilin-actin rods and Hirano bodies (Kang and Woo, 2019; Wurz et al., 2022). The stress conditions that promote rod formation impact cellular ATP levels; as a result, actin, an ATP-binding protein, shifts to a primarily ADP-bound state. The affinity of various actin-binding proteins, including cofilin, is higher for actin in its ADP-bound form and is one of the key factors that favor rod formation. While many aspects of cofilin-actin rod formation are known, the contributions of actin’s individual nucleotide-binding residues to ATP/ADP binding and subsequent cofilin-actin rod formation are poorly characterized. This is a critical knowledge gap, as numerous ATP-binding residues in the actin-ATP interface are associated with neurodegenerative diseases through either genetic mutation or post-translational modification (Varland et al., 2019).

Previously, we introduced the CofActor optogenetic system, which incorporates cofilin and actin and enables investigation of their interaction in a light- and stress-gated clustering response (Bunner et al., 2021; Salem et al., 2020). Specifically, CofActor consists of a blue-light responsive cryptochrome 2 (Cry2)-Cofilin.S3E protein fusion and a betaActin-CIB protein fusion and responds to stress stimuli, such as ATP depletion, that also induce native cofilin-actin rods. This prior work demonstrated that an ATP-bound Serine (Ser14) mutation could dramatically alter actin subcellular localization and the prevalence of anomalous actin structures, such as cofilin-actin rods, while enabling cofilin binding under non-stress conditions. This discovery supports the hypothesis that changes to residues [via genetic mutations or post-translational modifications (PTMs)] in the nucleotide-binding site of actin could be the basis for anomalous cytoskeletal structures associated with human disease. While S14V actin provides evidence for this possibility, numerous other residues potentially critical for ATP binding have yet to be investigated. A fuller characterization of the ATP binding site is essential to fully define the contributions of nucleotide-binding residues under both stress- and non-stress conditions to cytoskeletal anomalies. In this work, we probe the actin-nucleotide interface within the context of the CofActor optogenetic switch.

**FIGURE 1:**
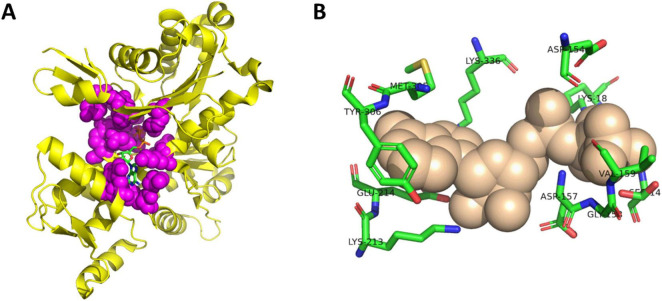
Structure of actin-ATP complex. **(A)** Crystal structure of actin bound to ATP (RCSB PDBID 1J6Z). Actin-ATP interface residues shown in purple spheres and ATP shown as sticks. **(B)** Detail of selected ATP-binding residues from actin-ATP interface. ATP shown as tan spheres and residues shown as sticks. Image created with PyMol.

**FIGURE 2:**
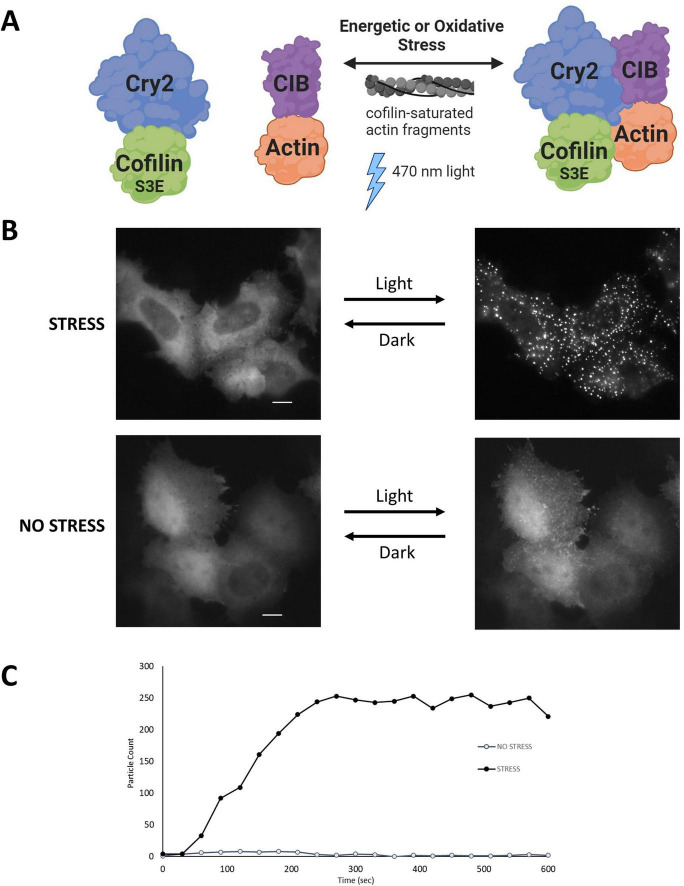
The CofActor (Cofilin Actin optically responsive) system. **(A)** CofActor consists of Cryptochrome2 (residues 1–498)—mCherry—Cofilin.S3E and beta Actin—CibN—GFP. Cytosolic cluster formation is promoted by blue light activation in the presence of oxidative or energetic stress, as shown in **(B)**. The interaction is reversible in the dark. **(C)** A time course of CofActor clusters formed post-light activation in HeLa cells with 470 nm light activation and imaging every 30 s. Particle counts were assessed with the FIJI/ImageJ Analyze Particles feature. Image in panel A created with BioRender.

To investigate the role of the actin nucleotide binding site in cofilin-actin rod formation, point mutations were made nucleotide-binding to residues within the actin-ATP binding site of the CofActor system (Figure 1A). These residues were selected for either their direct interaction with ATP or proximity to ATP in crystal structures of ATP-bound actin and are described in Table 1. Several of these residues (G15, G158, D157, R183, H73, M305) have been previously characterized in studies of actin-ATP binding and actin dynamics (Belmont et al., 1999; Müller et al., 2012; Nyman et al., 2002; Oosterheert et al., 2023; Posern et al., 2004). This work is differentiated from these prior studies by focusing on the anomalous actin structures (such as cofilin-actin rods) that result from perturbation of the actin-ATP interface. In link with this overall aim, these mutants were investigated for their cytoskeletal phenotypes under homeostatic and energetic stress (ATP-depleted) conditions using CofActor (Figure 2). This optogenetic approach identifies which amino acids are critical for actin function, as evidenced by changes in actin-rich structures, and their importance for sensing cellular stress, as evidenced by changes in the CofActor response.

**TABLE 1:** Actin-atp residues selected for this study.

Actin mutant	ATP interaction site^ł^
S14V*	Phosphate tail
K18A	Phosphate tail
G158L	Phosphate tail
V159L*	Phosphate tail
D154A	Phosphate tail
G15L/K	Phosphate tail
D157A	Ribose ring
K213A	Ribose ring
E214A/L	Ribose ring
Y306A	Adenosine
K336A	Adenosine
M305A/L	Adenosine
Y69A	Indirect interaction
E72A	Indirect interaction
S33A	Indirect interaction
R183A	Indirect interaction
H73A	Indirect interaction

*Analyzed in previous work (Salem et al., 2020).

^ł^Residues color coded by ATP-binding region.

This investigation identifies a subset of mutants (K18A, D154A, G158L, S14V, and K213A) that exhibit strikingly different cytoskeletal phenotypes under both homeostatic and stress conditions. These mutants do not respond to CofActor-mediated light activation in the same light- and stress-gated manner as wild-type actin, and their aberrant phenotypes are consistent in both immortalized and primary neuron cultures. Promisingly, these mutations could provide insight into the roles of select nucleotide-binding actin residues in the promotion of anomalous cytoskeletal features associated with neurodegenerative disease.

## Results and discussion

**FIGURE 3:**
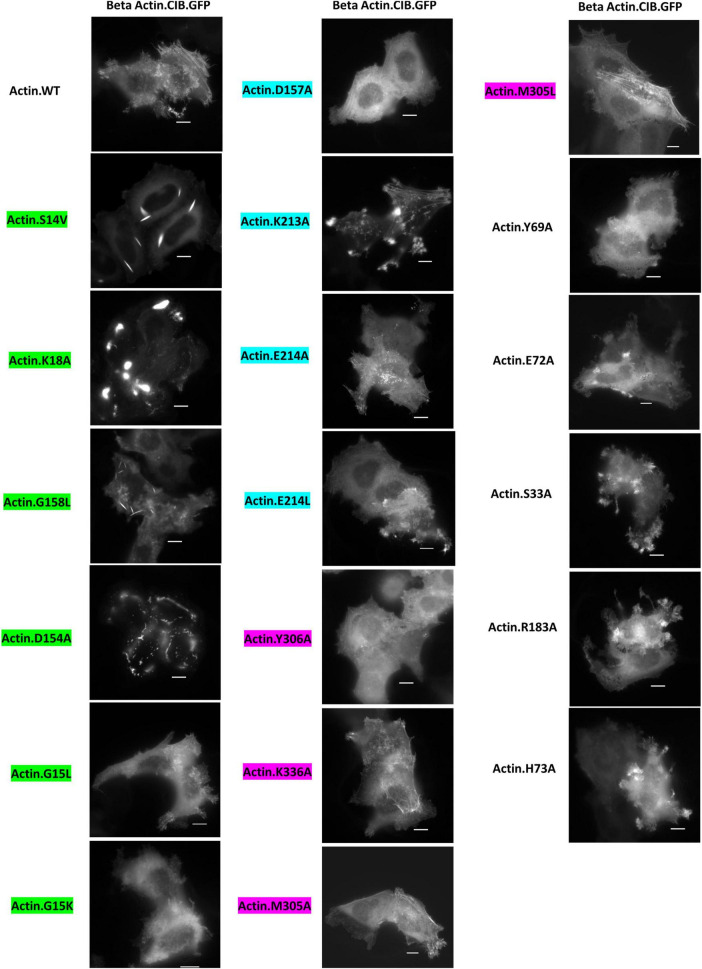
Distribution of Actin.CIB.GFP WT and mutant constructs in HeLa cells. Mutants G158L and S14V exhibit a cofilin-actin rod phenotype, while mutants K18A, D154A, and K213A exhibit an inclusion phenotype. Scale bars = 10 microns. Mutants are color-coded to correspond to their interaction with the phosphate tail (green), ribose ring (teal), or adenosine (magenta) of actin-bound ATP.

**FIGURE 4:**
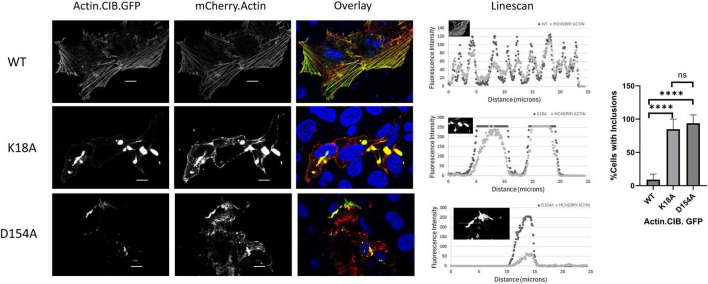
Actin mutants incorporate wild-type actin and disrupt actin-rich structures. HeLa cells expressing Actin.CIB.GFP (WT or mutant) and wild-type Actin.mCherry were fixed and imaged on a confocal microscope. Scale bars = 10 microns. Statistical analysis performed with One-way ANOVA (****p < 0.0001; n.s. = not significant).

For our initial studies of actin mutants, we examined the appearance of actin.CIB.GFP fusions in HeLa cells via widefield microscopy (Figure 3) to identify actin mutants with anomalous actin distributions. Mutant actin phenotypes were diverse: two mutants (G158L and the previously reported S14V) exhibited cofilin-actin rod phenotypes; three mutants (K18A, D154A, K213A) exhibited large inclusion phenotypes, with K18A and D154A having the most atypical subcellular distributions. We note that these large actin clusters are evocative of Hirano bodies, which are large actin-containing aggregates often associated with neurodegenerative diseases (Galloway et al., 1987; Maciver and Harrington, 1985; Mitake et al., 1997; Spears et al., 2014). To a lesser extent, the mutants S33A, E72A, H73A, R183A, and E214L also had atypical phenotypes, including localization to peripheral actin structures such as lamellopodia (Supplementary Figure 1). The remaining 11 mutants exhibited largely normal actin phenotypes (i.e., similar to WT actin), exhibiting a combination of cytosolic actin, stress fibers, and peripheral actin (results summarized in Table 2). Notably, the mutants with the greatest impacts on actin subcellular distribution were those involved in direct interactions with the phosphate tail of ATP. As these mutants disrupt a highly specific network of interactions with ATP, and the nucleotide-binding state of actin may contribute to changes in overall actin conformation (Reisler and Egelman, 2007), this result is consistent with the current understanding of actin structure. Interestingly, actin mutants with large inclusion phenotypes (K18A and D154A) also impact wild-type actin distribution (Figure 4), indicating that these mutants have a sequestering effect on non-mutant actin, increasing the likelihood that their presence will impact critical actin-dependent processes. The mutants also expressed at levels similar to the Actin.WT.CIB.GFP fusion (Supplementary Figure 2), implying that differences in actin structure, and not protein expression levels, are driving the observed anomalous cytoskeletal phenotypes. In addition, the Actin.WT.CIB.GFP construct exhibits a subcellular distribution similar to that of endogenous actin (Supplementary Figure 3). As such, it is anticipated that this construct is a reliable reporter of the cell’s overall actin distribution.

**TABLE 2:** Summary of actin mutant localization.

Actin mutant	Localization Pattern (typical or atypical)^ł^
WT	Typical
S14V*	Atypical (rods)
K18A	Atypical (inclusions)
G158L	Atypical (rods)
V159L*	Typical
D154A	Atypical (inclusions)
G15L	Typical
G15K	Typical
D157A	Typical
K213A	Atypical (inclusions)
E214A	Typical
E214L	Atypical (peripheral inclusions)
Y306A	Typical
K336A	Typical
M305A	Typical
M305L	Typical
Y69A	Typical
E72A	Atypical (peripheral inclusions)
S33A	Atypical (peripheral inclusions)
R183A	Atypical (peripheral inclusions)
H73A	Atypical (peripheral inclusions)

*Analyzed in previous work (Salem et al., 2020).

^ł^Residues color coded by ATP-binding region.

**FIGURE 5:**
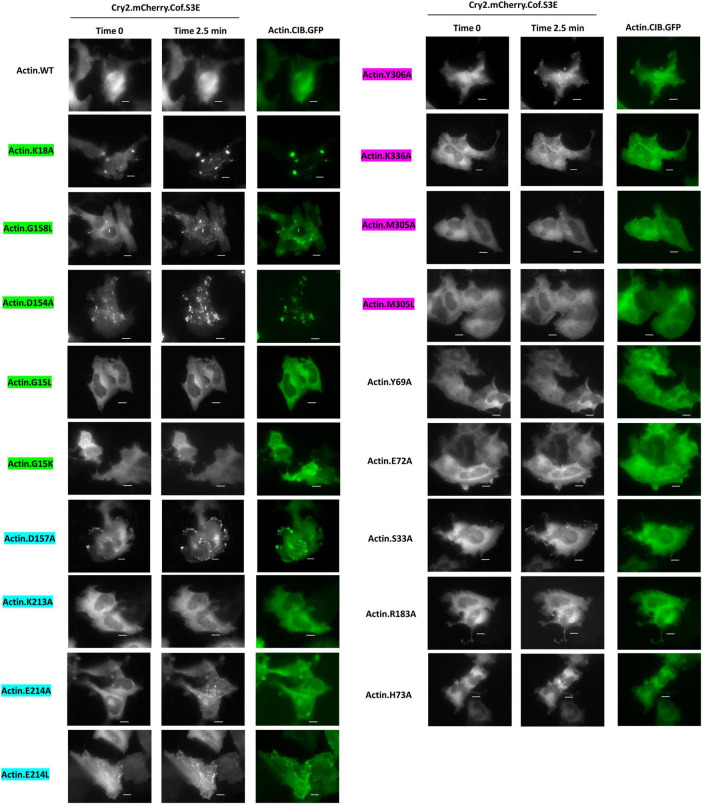
Cofilin recruitment to actin mutants in the absence of cellular stress. HeLa cells expressing Cry2.mCh.Cof and Actin (WT or mutant as indicated). CIB.GFP were imaged at 37°C in Dulbecco’s PBS. Images shown were taken before (Time 0) and post (Time 2.5 min) 470 nm light activation. Image exposures were set at 50 ms (GFP, 470 nm) and 200 ms (mCherry, 550 nm), with LED light sources at 50% power, and images were acquired every 30 s. Scale bars = 10 microns. Mutants are color-coded to correspond to their interaction with the phosphate tail (green), ribose ring (teal), or adenosine (magenta) of actin-bound ATP.

Next, using the CofActor system, we investigated whether the mutants responded to light activation in the presence of non-stress and energetic stress conditions in the same manner previously observed with wild-type Actin.CIB.GFP (low incorporation under non-stress and high incorporation under energetic stress conditions) (Salem et al., 2020). In the absence of energetic stress, light-activated recruitment of Cry2.mCh.Cofilin.S3E to mutant Actin.CIB.GFP was observed in those mutants that promoted a cofilin-actin rod phenotype or an aberrant clustering phenotype (Figure 5). These mutants include K18A, D154A, and G158L. We note that due to the significant pre-organization of these actin mutants in rods or inclusions, there is a high degree of light-independent pre-association of the Cry2.mCh.Cof reagent with these actin mutants, further distinguishing them from wild-type actin. By contrast, other mutants (ex. Y306A, S33A, E214L) with less dramatic alterations in their actin phenotypes nonetheless exhibited significant light-activated recruitment to CofActor clusters without applied cellular stress. These residues could, therefore, represent important mediators of the ATP-associated stress response. For example, light activation of the Y306A mutant in the absence of ATP depletion formed rods in ∼50% of cells analyzed (Figure 5). In contrast with the inclusion forming mutants (K18A, etc.), these rods were not the result of Cry2.mCh.Cof recruitment to pre-formed actin-rich structures. Thus, Y306A actin could also have utility as a stress-independent initiator of cofilin-actin rod formation within the optogenetic CofActor framework.

**FIGURE 6:**
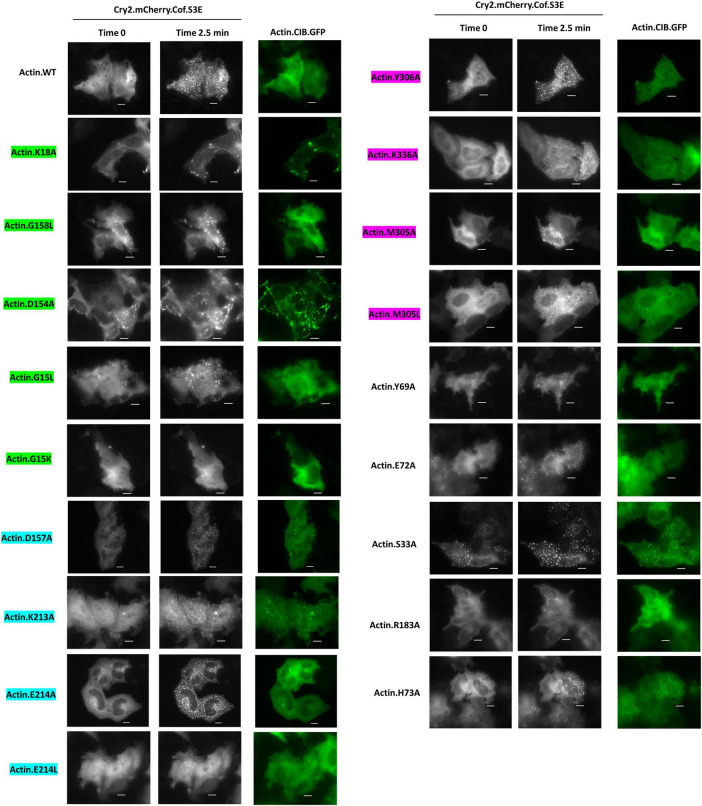
Cofilin recruitment to actin mutants in the presence of cellular stress. HeLa cells expressing Cry2.mCh.Cof and Actin (WT or mutant as indicated). CIB.GFP were imaged at 37°C in Dulbecco’s PBS with 6 mM 2-deoxy-D-glucose and 10 mM sodium azide. Images shown were taken before (Time 0) and post (Time 2.5 min) 470 nm light activation. Image exposures were set at 50 ms (GFP, 470 nm) and 200 ms (mCherry, 550 nm), with LED light sources at 50% power, and images were acquired every 30 s. Scale bars = 10 microns. Mutants are color-coded to correspond to their interaction with the phosphate tail (green), ribose ring (teal), or adenosine (magenta) of actin-bound ATP.

**FIGURE 7:**
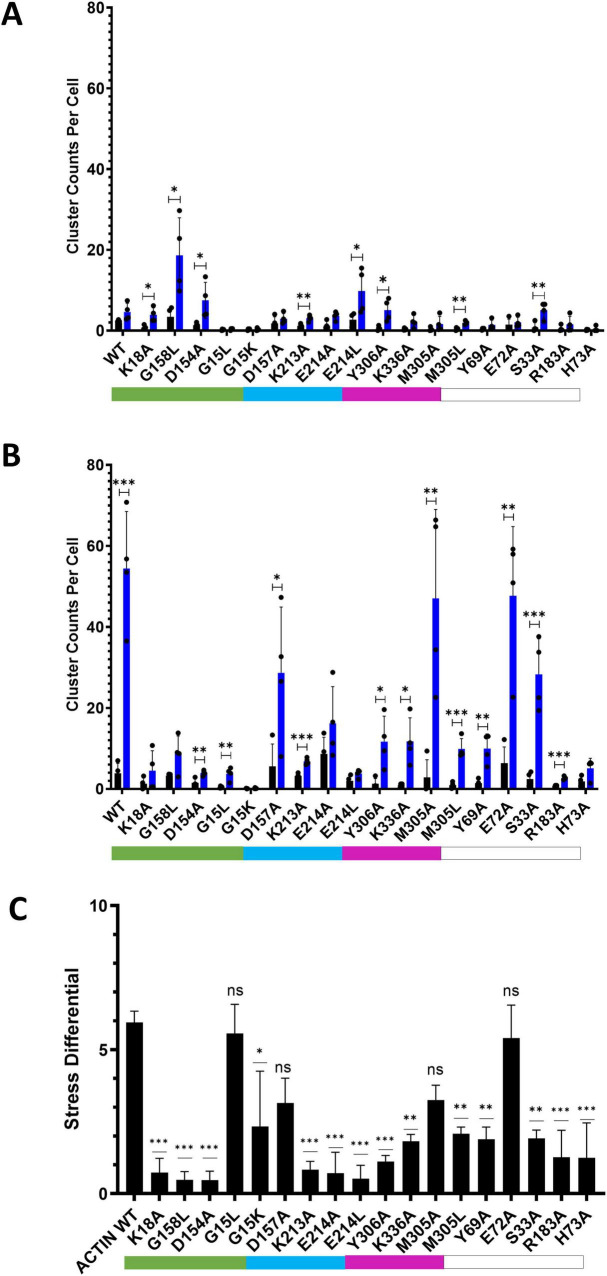
Comparison of CofActor WT and mutant light-activated cluster formation under non-stress and stress conditions. **(A)** Clustering under non-stress conditions after 5 min light activation (one 50 ms pulse every 30 s). **(B)** Clustering under stress (NaN3/2-DG) conditions (one 50 ms pulse every 30 s). **(C)** Stress differential (difference in 0 vs. 5 min fold changes between stress and non-stress conditions). Cluster counts were taken at times 0 (black bars) and 5 min (blue bars). Cluster counts represent the average of four replicate measurements (each measurement from a group with *n* ≥ 4 cells); error bars represent standard deviations **(A,B)** or propagated standard error. In **(C)**, constructs labeled ‘ns’ are statistically similar to the wild-type response. For **(A,B)**, pair-wise *t*-tests were used to determine statistical significance. ***p ≤ 0.001; **p ≤ 0.01;*p ≤ 0.05. For **(C)**, One-way ANOVA was used to determine statistical significance (ns = not significant). Color coded bars indicate direct contact with the ATP phosphate tail (green), ribose ring (teal), or adenosine ring (magenta).

When subjected to energetic stress (ATP-depletion conditions (10 mM NaN_3_, 6 mM 2-deoxy-D-glucose in Dulbecco’s PBS) previously shown to induce cofilin-actin rod formation (Bunner et al., 2021; Minamide et al., 2010; Salem et al., 2020; Figure 6), most actin mutants exhibited some degree of stress-promoted clustering with the exception of one non-responsive mutant (G15K). Four mutants (S33A, E72A, D157A, M305A) exhibited a similar degree of light-activated cluster formation (in terms of the number of clusters/rods formed per cell) as WT actin (Figures 7A,B), while the remaining 14 mutants exhibited compromised stress responses relative to WT actin.

To compare the various mutant responses under both non-stress and stress conditions, we applied a metric (the “stress differential,” Figure 7C) that compares the ratio of fold changes in light-activated cluster formation under both experimental conditions. This metric reveals which mutants exhibit responses closest to wild-type and which show little discrimination between stress and non-stress conditions (i.e., similar fold changes under both conditions result in a stress differential closer to one). By this metric, 4 mutants (G15L, E72A, M305A, and D157A) had a stress differential statistically equivalent to WT actin. It is anticipated that some of these mutants could have various stress-sensing applications and may present opportunities for further improving the orthogonality of the CofActor system. Interestingly, stress differential values of less than 1 were found for rod and inclusion-forming mutants (K18A, D154A, and G158L), indicating they were less responsive to light activation in the presence of cellular stress. ATP-depletion may result in further compaction of these actin inclusions, making them even less accessible to binding the Cry2.mCherry.Cof reagent. Overall, “loss of response” stress differentials were more prevalent among mutations to amino acids in direct contact with ATP versus those with indirect contacts.

**FIGURE 8:**
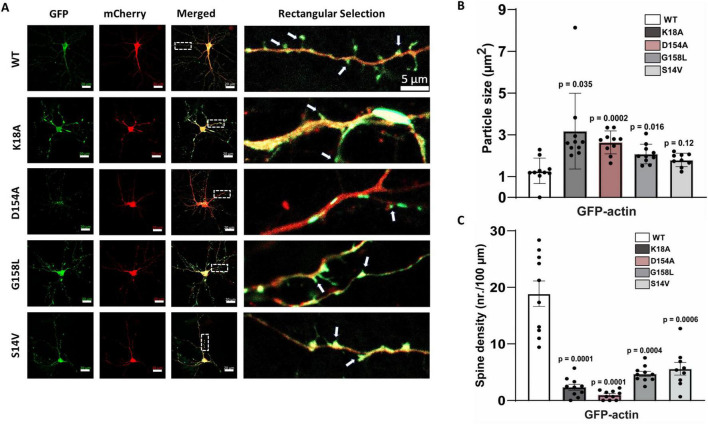
Actin expression and distribution in cortical neurons. **(A)** *On the left:* Representative images of dissociated cortical neurons transfected co-transfected with GFP-labeled wild-type actin or GFP-labeled actin mutants and mCherry (Red). Scale bars are 20 μm. The box represents the zoomed dendritic area, as shown on the *right* (scale bar is 5 μm; arrows indicate dendritic spines). **(B)** Graph shows average inclusion size for each mutant compared to neurons transfected with wild-type actin (9–10 neurons/condition; from 7 independent cultures). **(C)** Spine density for wild-type and mutant actin-expressing neurons. Error bars indicate S.E.M. One-way ANOVA was used to determine statistical significance and *p* ≤ 0.05 was considered significant.

These results demonstrate the importance of the specific ATP-amino acid contacts that maintain structural integrity of the actin-nucleotide binding pocket. Specifically, this integrity is important for regulating the actin stress response, which can be dramatically changed when key residues are missing or altered. The nucleotide-binding network of actin is highly conserved, and therefore, any alterations to this pocket can significantly impact actin phenotype and function (Pollard, 2016). However, the extent to which these phenotypes are generalizable, from immortalized to highly specialized cell types, is unknown. As a result, we subsequently asked whether the strong actin clustering phenomena associated with a subset of mutants would translate into cortical neurons. We investigated the actin distribution of K18A, G158L, D154A, and S14V compared to WT actin within the Actin.CIB.GFP fusion (Figure 8; Supplementary Figure 4). Similar to HeLa cells, these mutants were highly aggregated in neurons, indicating that they might provide insight into the role of ATP-binding inhibited actin in the neurological disease state (Figure 8A). Quantitation of mutant clustering versus WT actin (Figure 8B) reveals a statistically significant increase in inclusion size. This effect was most pronounced in neurons expressing the D154A and K18A mutants and, to a lesser extent, with G158L and S14V. Next, we analyzed spine density differences between the WT actin and mutant actin-expressing neurons (Figure 8C). The actin mutants with increased clustering (K18A, D154A) led to a significant reduction in the number of spines compared to WT actin-expressing neurons. This data suggests that the aggregated actin in the mutant-expressing neurons leads to structural destabilization of dendritic spines, resulting in spine elimination and/or defective spinogenesis. We argue that these mutations could be responsible for sequestration effects, such as the binding up of WT actin and actin-binding proteins, that disrupt neuronal cytoskeletal dynamics. Such effects provide a link to neurodegenerative diseases and could be further exploited by using these mutants to screen for drugs that reverse these anomalous structures.

In conclusion, several actin mutants that produce atypical morphologies, ranging from rods to large inclusions, were identified in this study. We propose that mutants that accumulate into rods or inclusions may lack the ability to bind ATP efficiently. For example, the mutant G158L formed rods similar to endogenous cofilin-actin rods. The change from glycine to a large hydrophobic amino acid may impact nucleotide binding by occluding ATP from the binding pocket to achieve an ADP-actin-like conformation, thus promoting inclusion into cofilin-actin rods. The mutants K18A and D154A, which form large actin inclusions, also indicate the criticality of specific contacts between the nucleotide-binding pocket and ATP. In these cases, the compromised ATP-binding may result in unregulated actin accumulation into large inclusions. In the case of K213A, the absence of a strong stress-induced light response may be due to the loss of a critical interaction between the amino acid and the ribose ring of the ATP, thus decoupling actin from sensitivity to its stress associated nucleotide-bound state.

Some of the mutants investigated have been identified in prior studies of post-translationally modified residues in actin (Varland et al., 2019). These include residues K18, Y306, and K213 (Table 3). Specifically, actin undergoes post-translational modification in response to cellular stress. These modifications (methylation, acetylation, nitrosylation, ubiquitination) can effectively disable residues within the ATP binding site, leading to aberrant actin structures (Varland et al., 2019). Further investigation of these actin mutants may provide insight into the role of post-translationally modified actin in cellular stress response, synaptic loss, and cell death. In particular, post-translational modification of residue K18 may be relevant to neurodegenerative diseases (Table 4), as our data indicates that mutation of K18 results in a Hirano body-like phenotype.

**TABLE 3:** Actin mutants from this study associated with neurodegenerative disease.

Mutation	Effect	Disease relevance^a, b, c, d^
K18	Large actin inclusions	Hirano bodies: Alzheimer’s disease; Frontotemporal dementia; Creutzfeldt–Jakob disease
S14V	Cofilin-actin rods	Alzheimer’s, Parkinson’s, and Huntington’s diseases
G158L	Cofilin-actin rods	Alzheimer’s, Parkinson’s, and Huntington’s diseases

**^a–d^** Galloway et al. (1987); Maciver and Harrington (1985), Bamburg and Bernstein (2016), Maloney and Bamburg (2007).

**TABLE 4:** Actin residues from this study associated with confirmed PTMs^a^.

Residue	Post-Translational Modification^a^
K18	Methylation, Ubiquitination
Y306	Phosphorylation
K213	Acetylation, Ubiquitination

^a^Varland et al. (2019)

In the future, we will investigate photocaging strategies for actin inclusion-forming mutants such as K18A. In this strategy, light-activated photocaging domains will be used to prevent pre-accumulation of these actin mutants into rod-like structures in the dark. This approach could result in a single-component actin-based photoswitch that could be used to study the effect of introducing aberrant cytoskeletal dynamics under a wide array of experimental conditions.

### Experimental procedures

#### Plasmids and cloning

Cloning of the β-Actin.Cib.GFP construct and the Cry2PHR.mCh.Cof construct was conducted according to previously reported methods (Bunner et al., 2021; Salem et al., 2020). Briefly, genes encoding β-Actin and Cib were PCR amplified with overlapping primer sequences. Gene fragments were gel isolated and stitched together via splice overlap extension (SOE) PCR. The resulting gene fragment was trimmed via restriction digest, followed by ligation into complementary restriction sites in the target plasmid. Point mutations were introduced into the gene encoding Actin.Cib.GFP in a phCMV-GFP plasmid (Genlantis) plasmid using mutagenic primers (IDT DNA) following a standard protocol for site-directed mutagenesis. mCherry-Actin-C-18 was a gift from Michael Davidson (Addgene plasmid # 54967) (Rizzo et al., 2009). mCherry was a gift from Rob Parton (Addgene plasmid # 176016) (Michael et al., 2016).

#### Cell lines and transfection

Midi prep quantities of DNA of each construct were created from *E. coli* and collected for cell transfection. Transfection of HeLa cells was then performed with Calfectin reagent (SignaGen) following the manufacturer’s suggested protocols. Briefly, for dual transfections in 35 mm glass bottom dishes, plasmid DNA was combined in a 1:1 ratio (1,250 ng per plasmid for dual transfections) in 100 μL of DMEM, followed by the addition of 3 μL of Calfectin reagent. The solution was incubated at room temperature for 10 min, followed by dropwise addition to cell culture. Transfection solutions were allowed to remain on cells overnight. Cells were maintained at 37°C and 5% CO_2_ in a humidified tissue culture incubator in a culture medium of DMEM supplemented with 10% FBS and 1% Penicillin-Streptomycin.

##### Live cell experiments

Transfected HeLa cells were washed with Dulbecco’s PBS (with calcium and magnesium; 3 × 1 mL) before treatment with ATP depletion medium (6 mM D-Deoxyglucose and 10 mM Sodium Azide in Dulbecco’s PBS) or DPBS for 15 min before imaging on a widefield microscope.

#### Mouse cortical neuron cultures and transfection

Dissociated cortical neuron cultures were prepared using a previously described protocol modified for E18 embryonic cultures (Bessetti et al., 2025). Briefly, the CD1 pregnant mouse was euthanized with CO_2_ according to an East Carolina University IACUC-approved protocol. The abdomen was cleaned with 70% ethanol, followed by an incision in the middle of the abdomen to expose the uterine horns containing the embryos. The uterus was immediately transferred to a 100 mm dish containing ice-cold PBS. Single embryos were collected and transferred into a new 100 mm dish with ice-cold PBS to remove blood and embryonic fluid. The embryo heads were then cut and transferred into a new 100 mm dish containing an ice-cold dissection medium, prepared as described (Chang et al., 2017). The brain from each embryo head was gently removed, followed by dissection to isolate the cortices under a dissection stereoscope. Cortices were washed twice with ice-cold dissection medium, then digested and dissociated using Neuronal Isolation Enzyme using the manufacturer’s protocol (ThermoFisher). Dissociated neuron cultures were plated in culture medium containing BME (Basal Medium Eagle) supplemented with 10% BCS and 1% Penicillin-Streptomycin at a density of 500,000 cells/well into 12-well plates containing glass coverslips coated with PDL/Laminin. On the day *in vitro* 2 (DIV2), the culture medium was changed to Neurobasal/B27 plus culture medium, supplemented with Glutamax and 1% Penicillin-Streptomycin, which was changed twice weekly. Neurons were transfected with the GFP-tagged actin mutant constructs and mCherry (6 μg plasmid/well) on DIV5 using Lipofectamine LTX reagent (Invitrogen).

##### Fixed neuron experiments

Forty eight to seventy two hours post-transfection, the culture medium was removed, and neurons were fixed for 20 min with pre-warmed 4% Paraformaldehyde solution in 0.1 M PBS (37°C; prepared from 16% PFA; Electron Microscopy Sciences) at room temperature. Coverslips were washed with PBS (2 × 20 min) and mounted on glass microscope slides using ProLong™ glass antifade mountant with NucBlue™ stain (Invitrogen).

##### Confocal microscopy

Confocal images of fixed cells were obtained with an Olympus IX2-DSU tandem spinning disk confocal laser scanning microscope or with a Zeiss LSM 800 microscope with Airyscan technology. Fluorescence images were colorized and overlaid using FIJI software.

##### Widefield microscopy

A Leica DMi8 Live Cell Imaging System, equipped with an OKOLab stage-top live cell incubation system, LASX software, Leica HCX PL APO 63x/1.40–0.60na oil objective, Lumencor LED light engine, CTRadvanced + power supply, and a Leica DFC900 GT camera, was used to acquire images. Exposure times were set at 50 ms (GFP, 470 nm) and 200 ms (mCherry, 550 nm), with LED light sources at 50% power, and images were acquired every 30 s over a 10-min time course.

#### Particle counting

Analysis of imaging data was performed in FIJI, equipped with the BioFormats package. Particle counting was performed using the Analyze Particles feature. For HeLa cell experiments, particle size was restricted from 20 to 200 pixels, and circularity was restricted from 0.2 to 1.00 μm. Particle counts are reported as the average particles per cell. For neuron experiments, particle size was restricted to 1–10 μm, and circularity was restricted to 0.2–1.00 μm. Particle areas are reported as the average particle area (μm^2^) per cell.

Spine density was determined using FIJI ImageJ software. For each neuron, five different secondary dendrites were randomly selected to ensure unbiased measurements. The perimeter of each dendrite was measured and used to calculate its length. Individual dendritic spines, defined as small bulbous protrusions of the dendrites, were manually counted using the zoom-in feature of ImageJ. Filopodia were not counted. The density of dendritic spines was then calculated by dividing the total number of spines by the length of the dendrite in microns.

#### Western blotting

HeLa cells were lysed post-transfection with 200 μL of M-PER lysis buffer (Thermo Scientific) plus protease inhibitors. After 10 min on a rotary shaker at room temperature, lysates were collected and centrifuged for 15 min (94 rcf; 4°C). The supernatants were combined with Laemmli sample buffer and used for further experiments. The resulting lysates were subjected to electrophoresis on a 10% SDS-PAGE gel and then transferred onto PVDF membranes (20 V, overnight, at 4°C). Membranes were then blocked for 1 h with 5% BSA in TBS with 1% Tween (TBST), followed by incubation with primary antibody (anti-GFP antibody (Santa Cruz #sc-8334); 1:1,000 dilution in 5% BSA – TBST) overnight at 4°C on a platform rocker. The membranes were washed 3 × 5 min each with TBST and incubated with the appropriate secondary antibody in 5% BSA—TBST for 2 h at room temperature. After washing 3 × 5 min with TBST, the membranes were exposed to a chemiluminescent substrate for 5 min and imaged using an Azure cSeries imaging station.

#### Statistical analyses

Statistical significance (*p*-values) was determined using SigmaPlot v. 13.0 (Systat Software, Inc.). *T*-tests were performed to analyze particle count data in HeLa cells. One-way ANOVAs (Holm-Sidak method) were used to analyze stress differentials in HeLa cells and particle size data in neurons (Holm, 1979). Plots were constructed using Graph Pad Prism version (8.2.1).

## Data Availability

The raw data supporting the conclusions of this article will be made available by the authors, without undue reservation.
