# Supplementary material for: Identification of actin mutants with neurodegenerative disease-like phenotypes via mutagenesis of the actin-ATP interface
Source: Front Cell Neurosci. 2025 Jun 4;19:1543199. doi: 10.3389/fncel.2025.1543199 (PMC12174402; doi:10.3389/fncel.2025.1543199)
Supplement: Supplementary file 1 [file Data_Sheet_1.PDF]

SUPPORTING INFORMATION

FOR

Identification of actin mutants with neurodegenerative disease-like phenotypes via mutagenesis of the actin-ATP interface

Noah Mann[1]*, Keerthana Surabhi[2]*, Josephine Sharp[1], Mary Phipps[2], Maelee Becton[2], Jahiem Hill[1], Davis Roberts[1], Erzsebet Szatmari[2]**, Robert Hughes[1]**

1. Department of Chemistry, East Carolina University, Greenville, NC 27858 2. Department of Physical Therapy, East Carolina University, Greenville, NC 27858.

*co-first authors, contributing equally

**co-corresponding authors

CONTENTS

SUPPORTING FIGURE 1

SUPPORTING FIGURE 2

SUPPORTING FIGURE 3

SUPPORTING FIGURE 4

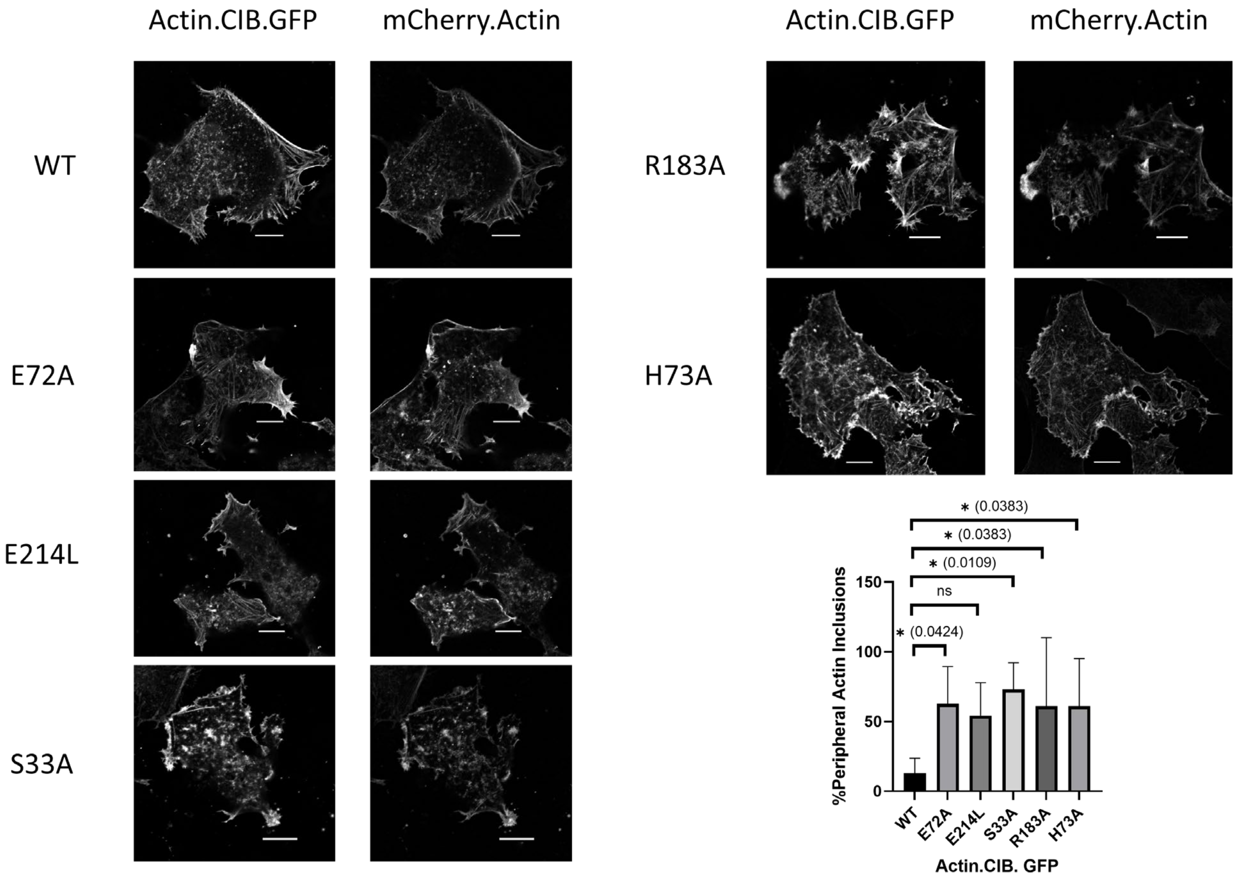

Supporting Figure 1. Actin mutants that promote peripheral actin inclusions. Actin mutants E72A, E214A, S33A, R183A, and H73A were observed to promote peripheral actin inclusions. One-way ANOVA analysis of cells (from n = 5 – 7 groups of 4 – 8 cells each) identified E72A, S33A, R183A, and H73A as having statistically significant (* $p < 0.05$) differences from WT actin. Images were acquired with a confocal microscope (Zeiss 900).

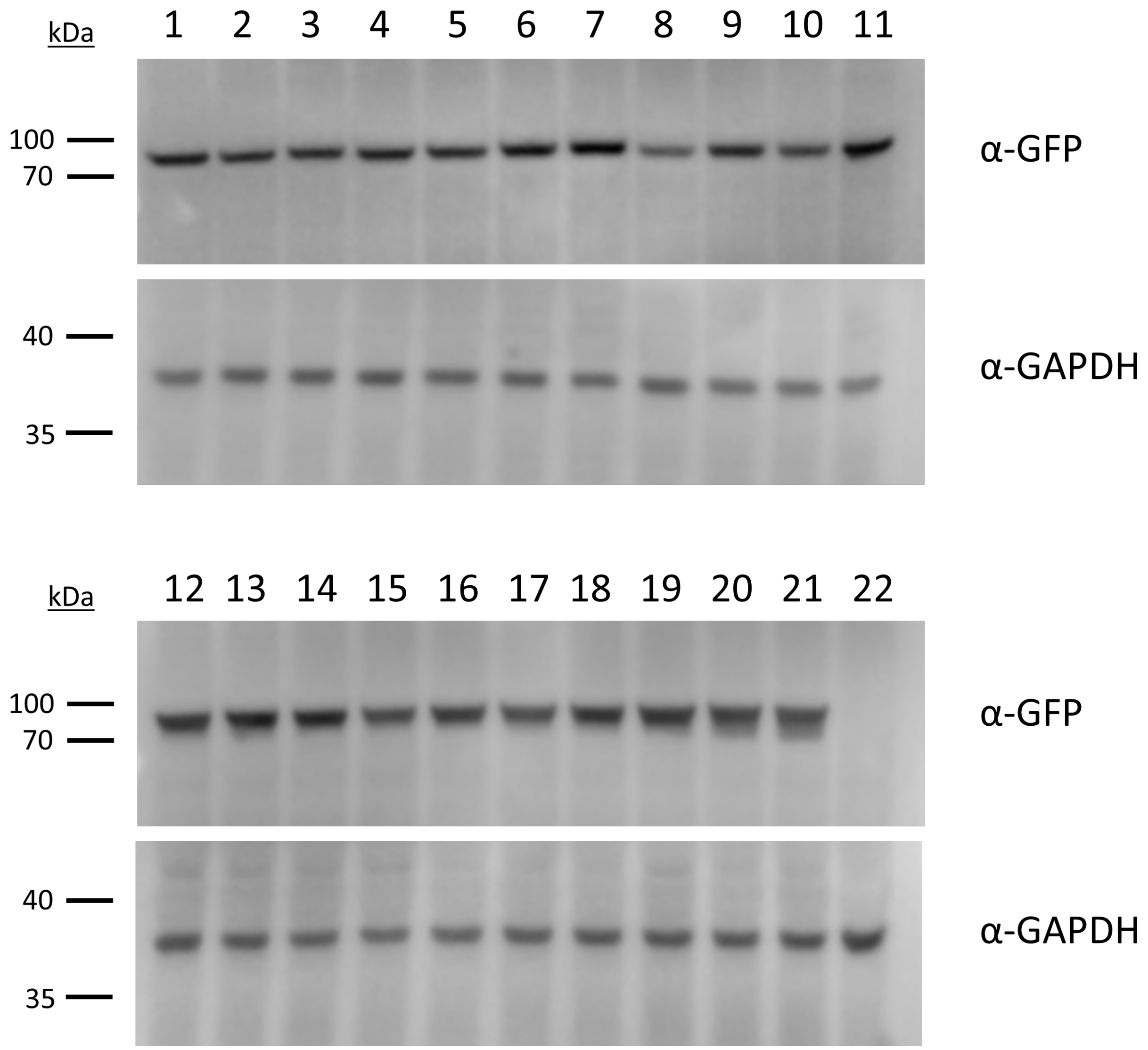

Supporting Figure 2. α-GFP western blot of actin mutant constructs from HeLa cell lysates. Key: 1. WT, 2. S14V, 3. K18A, 4. G158L, 5. V159L, 6. K213A, 7. Y306A, 8. D154A, 9. E214A, 10. E214L, 11. K336A, 12. D157A, 13. Y69A, 14. E72A, 15. S33A, 16. R183A, 17. H73A, 18. G15L, 19. G15K, 20. M305A, 21. M305L, 22. Non-transfected control

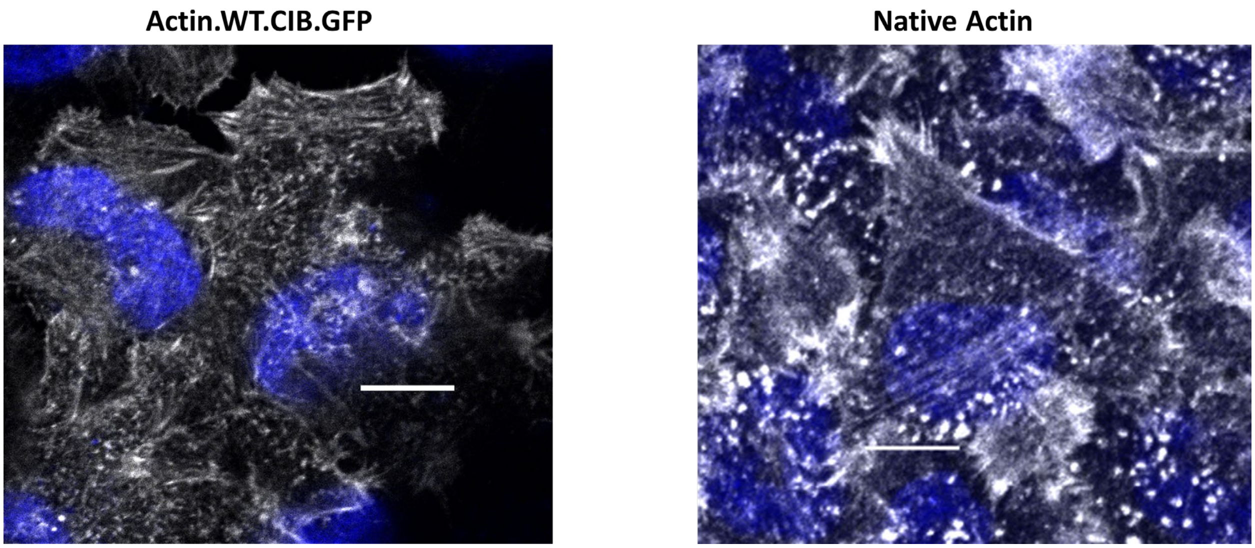

Supporting Figure 3. Comparison of Actin.CIB.GFP and native actin in HeLa cells. Cells were transfected with Actin.CIB.GFP (left) or not transfected (right) and fixed with 4% paraformaldehyde in DPBS. Non-transfected cells were subsequently immunostained for native actin (anti-Actin (Santa Cruz), 1:500 in antibody dilution buffer, overnight at 4 °C; Alexa 488 conjugated goat anti-mouse secondary (Invitrogen; 1:1000 in antibody dilution buffer, 1 hour at room temperature). The distribution of Actin.CIB.GFP resembles that of native actin distribution, with a mixture of stress fibers, small actin clusters, filopodia, and other peripheral actin structures.

Supporting Figure 4 (FOLLOWING PAGE). Side-by-side of Actin.CIB.GFP constructs and mCherry filler. Same as images shown in Figure 8 but with separate red and green channels.

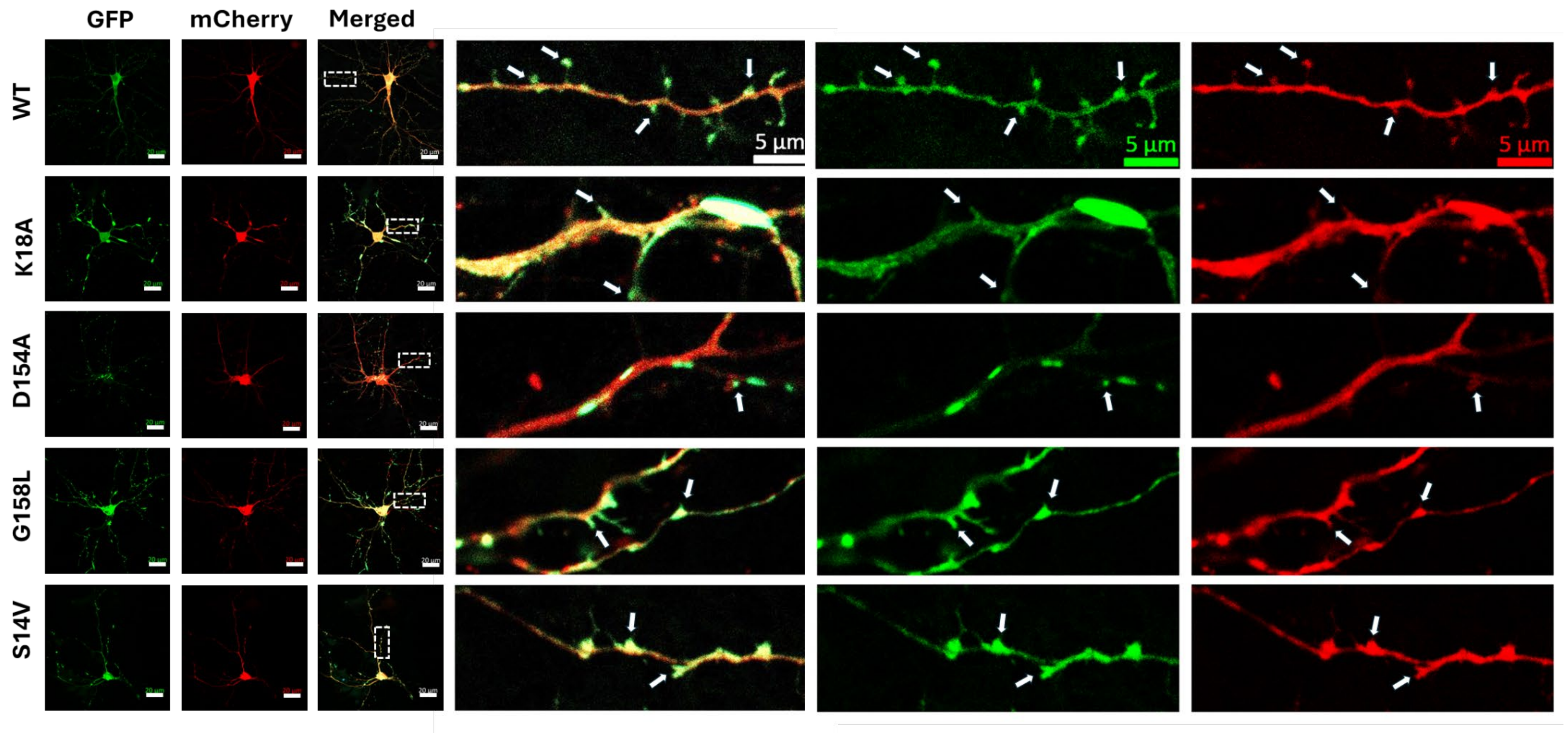

GFP
mCherry
Merged
WT
K18A
D154A
G158L
S14V
5 µm
5 µm
5 µm
